# Genomic and Antimicrobial Surveillance of *Campylobacter* Population in Italian Poultry

**DOI:** 10.3390/foods12152919

**Published:** 2023-07-31

**Authors:** Francesca Marotta, Anna Janowicz, Romina Romantini, Lisa Di Marcantonio, Federica Di Timoteo, Teresa Romualdi, Katiuscia Zilli, Lisa Barco, Mario D’Incau, Iolanda Mangone, Francesca Cito, Marco Di Domenico, Francesco Pomilio, Lucilla Ricci, Giuliano Garofolo

**Affiliations:** 1 National Reference Laboratory for Campylobacter, Istituto Zooprofilattico Sperimentale Teramo, Via Campo Boario 1, 64100 Teramo, Italy; f.marotta@izs.it (F.M.); a.janowicz@izs.it (A.J.); l.dimarcantonio@izs.it (L.D.M.); f.ditimoteo@izs.it (F.D.T.); t.romualdi@izs.it (T.R.); k.zilli@izs.it (K.Z.); i.mangone@izs.it (I.M.); f.cito@izs.it (F.C.); m.didomenico@izs.it (M.D.D.); f.pomilio@izs.it (F.P.); l.ricci@izs.it (L.R.); g.garofolo@izs.it (G.G.); 2 Italian National Reference Laboratory for Salmonellosis, Istituto Zooprofilattico Sperimentale delle Venezie, Legnaro, 35020 Padua, Italy; lbarco@izsvenezie.it; 3 Istituto Zooprofilattico Sperimentale della Lombardia ed Emilia Romagna “Bruno Ubertini”, 25124 Brescia, Italy; mario.dincau@izsler.it

**Keywords:** *Campylobacter*, poultry, antimicrobial resistance (AMR), multidrug resistance (MDR), resistance genes, virulence genes, whole-genome sequencing (WGS)

## Abstract

*Campylobacter* is one of the most common foodborne diseases worldwide with increasing rates of antibiotic resistance. Most cases of campylobacteriosis can be traced back to the consumption of poultry meat. Despite many efforts to reduce contamination in farms and in slaughterhouses, the persistence of this pathogen in poultry products remains a problem. This study aimed to evaluate the genetic diversity and antibiotic resistance of 542 *C. jejuni* and *C. coli* in Italian poultry, in the framework of two National Monitoring Programs. Genomes were screened for antibiotic resistance, virulence determinants and contextualized within a global collection of *C. jejuni*. ST2116, ST2863 and ST 832 were the most prevalent and significantly associated with Italian poultry. A worrying increase in resistance to quinolones, fluoroquinolones and tetracycline was observed in *C. jejuni*, while an increased occurrence of multidrug resistant (MDR) strains and strains resistant to macrolides was detected in *C. coli*. Low resistance rates were found for aminoglycosides. Molecular resistance determinants were consistent with the phenotypic resistance for tetracycline and quinolones. In silico analysis revealed 119 genes associated with virulence factors, with a notably higher prevalence of some genes in ST2863 genomes. This study highlights the increased resistance to macrolides and the emergence of MDR strains for *C. coli*, the genetic basis of AMR and the predominance of two genotypes among *Campylobacter* strains isolated from the Italian poultry farms.

## 1. Introduction

Foodborne disease caused by *Campylobacter* continues to be a major global problem worldwide and the spread of the multidrug resistant (MDR) strains makes clinical therapy challenging [1,2]. Since 2007, campylobacteriosis has been the most frequently reported gastrointestinal infection in humans in the EU [3]. In 2021, the number of confirmed cases of human campylobacteriosis was 127,840, corresponding to an EU notification rate of 41.1 per 100,000 of population. This was an increase of 2.1% compared with 2020 [3]. *C. jejuni* and *C. coli*, accounting for most human infections, can be found in animal gastrointestinal tracts and within the environment contaminated by manure [4]. In addition, some studies demonstrated the existence of stable associations between particular *Campylobacter* genotypes and different host sources [5]. Poultry is now recognized as the principal reservoir of *Campylobacter*, because broiler carcasses can be highly contaminated during the defeathering and evisceration process with chicken faeces containing high concentrations of bacteria (1.0 × 1010 CFU/g) [6]. As reported by Colles et al., humans currently consume around 60 billion chickens per year, far more than any other food of animal origin [7]. Attribution modeling studies estimated that between 58 and 78% of human cases of campylobacteriosis originate from contaminated chicken meat [5]. Considering that the broiler meat is the most important source of human infection, the prevention of broiler flock colonization is a food safety priority [8,9]. For this reason, the EU introduced a new regulation (amendment of Annex I to EC regulation No. 2073/2005 with regard to *Campylobacter* as a process hygiene criterion in broiler carcasses) in force since 2018. In the EU, the top five chicken meat-producing countries are Poland, the United Kingdom, Germany, France and Italy. Poultry meat consumption has increased in recent years in Italy, making it one of the most widely consumed meats in the country. Indeed, data reported in 2022 confirmed that Italy is a top EU country in terms of the poultry meat self-supply, resulting in overall self-sufficiency at 108% [10]. The clinical manifestation of campylobacteriosis in humans is usually a self-limiting disease and normally does not require antibiotic therapy. However, in patients with severe symptomatology, macrolides (erythromycin and azithromycin), fluoroquinolones (ciprofloxacin) and tetracycline are the most effective antibiotics used [11]. Unfortunately, many analogues to these antimicrobials are widely used in veterinary settings, leading to the emergence and circulation of resistant isolates of *Campylobacter*. A worrying increase in the multidrug-resistant (MDR) *Campylobacter* strains from farm to fork has been observed worldwide due to the continuous increase of resistance to the antimicrobials used in clinical practice and in the veterinary field [12]. Antimicrobial resistance determinants acquisition in *C. jejuni* and *C. coli* is the consequence of well-established mutations, such as the A2075G mutation in the gene encoding 23S rRNA or the C257T mutation leading to the T86I substitution in the gyrA gene, which are linked to macrolides and quinolones resistance, respectively [13,14]. Moreover, antimicrobial resistance can also be due to horizontal gene transfer and may be favoured in environments such as broiler farms and under selective pressure caused by the overuse of antimicrobials in farms [15]. 

The severity of campylobacteriosis depends on the infecting strain and on the immune status of the host. Although *Campylobacter* does not carry classical virulence factors such as enterotoxins, other determinants involved in survival and colonization, linked to motility, adhesion, invasion, capsule synthesis, cytolethal distending toxin synthesis, lipooligosaccharide (LOS) and secretion systems can determine the severity of the infection and *Campylobacter* persistence in the environment [16,17,18]. However, the virulence factors that cause clinical symptoms are not yet well understood [19]. 

Whole genome sequencing (WGS) has become an effective method for investigating the evolution of foodborne pathogens even over short time periods [20]. This powerful tool offers not only a high-resolution sub-typing of *Campylobacter* essential for outbreak investigation, but it may also predict antibiotic resistance [17,21,22,23]. Taking into consideration the increasing importance of *C. jejuni* and *C. coli* as foodborne pathogens, the aim of the current study was to investigate the occurrence and the distribution of *Campylobacter* in broiler chickens in Italy over the past 15 years, including the identification of potential virulence factors and antimicrobial resistance profiles. The results of this study provided insights into the features of *Campylobacter* in the Italian poultry production chain and highlighted the necessity of mitigating the risk of *Campylobacter* transmission to humans through food.

## 2. Materials and Methods

### 2.1. Experimental Design and Strains Selection

In this study, we analysed a collection of *C. jejuni* and *C. coli* collected in the framework of National Monitoring Programs carried out in 2008 and 2015. According to these surveillance plans, sampling was conducted in slaughterhouses distributed throughout the national territory representative of the most common Italian broiler production facilities [24]. The samples included caecal content and carcass skins collected after chilling. The first sampling, carried out according to the EU baseline study on broilers in 2008, included 48 slaughterhouses distributed in 11 different geographical sites, located in the north (4), center (4) and the south of Italy (3) [24], accounting for a total of 393 slaughter batches. The second one, carried out according to a National Monitoring Plan was a 1-year longitudinal study (2015–2016), and included 450 poultry batches collected throughout the entire national territory. A total of 35 slaughterhouses was included. In total, we selected 542 *Campylobacter* strains stored at the Italian National Reference Laboratory for *Campylobacter* (NRL, http://www.izs.it/IZS/Eccellenza/Centri_nazionali/LNR_-_Campylobacter, accessed on 14 March 2022). The collection included 63 *C. jejuni* and 7 *C. coli* strains of the EU baseline and 317 *C. jejuni* and 155 *C. coli* strains of the National Monitoring Plan. A total of 330 carcass skins and 212 caecal contents was examined. The selection of isolates to be included in the present study was based on the Italian poultry production and each region contributed with a number of isolates proportional to its production.

### 2.2. DNA Extraction and PCR Identification

The frozen stock stored at −80 °C in Microbank™ was cultured on Columbia blood agar plates and incubated at 42 °C microaerobically (10% CO_2_, 5% O_2_ and balancing N_2_) for 48 h in a controlled atmosphere incubator (ICO 240med, Memmert, Büchenbach, Germany). DNA was extracted with a Maxwell instrument (Promega Corporation, Madison, WI, USA) according to the manufacturer’s protocols. DNA concentration was quantified using a Nanodrop Spectrophotometer (Nanodrop Technologies, Celbio Srl., Milan, Italy). After an initial phenotypic characterization, a multiplex PCR was used to identify the colonies at species level, as described previously [25]. Strains used as positive controls were *Campylobacter jejuni* ATCC 33291, *Campylobacter coli* NCTC 11353, *Campylobacter lari* NCTC 11552, *Campylobacter fetus* ATCC 19438 and *Campylobacter upsaliensis* NCTC 11541. The dataset analysed in this study is detailed in Table S1.

### 2.3. Antimicrobial Susceptibility Tests

The broth microdilution method using a Sensititre automated system (TREK Diagnostic Systems, Venice, Italy) was used to determine the antimicrobial susceptibility of 380 *C. jejuni* and 162 *C. coli* strains, following the international guideline recommendations of the European Committee on Antimicrobial Susceptibility Testing (www.eucast.org, accessed on 14 March 2022). Six antibiotics were tested. Colonies were subcultured on Columbia agar for 24 h, seeded in Mueller Hinton Broth supplemented with blood (Oxoid, Basingstoke, UK) and then dispensed into EUCAMP2 plates (TREK Diagnostic Systems, Venice, Italy), according to the manufacturer’s instructions. These plates contained known scalar concentrations of the following antibiotics: ciprofloxacin (CIP) (0.12–16 μg/mL), erythromycin (ERY) (1–128 μg/mL), gentamicin (GEN) (0.12–16 μg/mL), nalidixic acid (NAL) (1–64 μg/mL), streptomycin (STR) (0.25–16 μg/mL), and tetracycline (TET) (0.5–64 μg/mL). After bacterial inoculation, the plates were incubated for 24 h at 42 °C microaerobically and then checked. *Campylobacter* was classified as resistant (R), and susceptible (S) according to minimum inhibitory concentration (MIC) breakpoints using Swin v3.3 Software (Thermo Fisher Scientific, Waltham, MA, USA), following the epidemiological cutoff values (ECOFFs) defined by EUCAST (the European Committee on antimicrobial breakpoints) (www.eucast.org, accessed on 14 March 2022). The *Campylobacter jejuni* strain NCTC 11351 was used as a control. Broth microdilution results are detailed in Table S1.

### 2.4. Whole Genome Sequencing and Genomic Characterization

All isolates were sequenced. DNA extractions were quantified by a Qubit 2.0 Fluorometer (Life Technologies, Carlsbad, CA, USA) and DNA libraries were prepared using a Nextera XT Library Preparation Kit (Illumina, Inc., San Diego, CA, USA) and then sequenced with an Illumina NextSeq 500 sequencer. Sequence reads (150-bp, pair-end) were demultiplexed and the adapters were removed and trimmed with a Trimmomatic tool (version 0.36) and reassembled using SPAdes version 3.11.1 with the ‘careful’ option selected (48). The sequence reads generated in this study were deposited in the NCBI Sequence Read Archive (SRA) in Bioprojects PRJNA941181 (http://www.ncbi.nlm.nih.gov/bioproject/941181, accessed on 14 March 2022) and PRJNA941194 (http://www.ncbi.nlm.nih.gov/bioproject/941194, accessed on 14 March 2022). *C. jejuni* and *C. coli* genome assemblies were genotyped by MLST and cgMLST. The MLST profiles were assigned using a *C. jejuni*/coli task template MLST 7 loci, accessible at https://pubmlst.org/Campylobacter/ available through Ridom SeqSphere+ V 6.0.2 software (RidomGmbH, Münster, Germany). Minimum spanning trees (MST) were created using default software settings. The assemblies were also investigated for the genomic AMR traits. The cgMLST profiles were assigned using a *C. jejuni*/coli task template with 637 target core genomes available in the Ridom SeqSphere+ database (https://www.ridom.de/seqsphere/u/Task_Template_Sphere.html, accessed on 14 March 2022). A minimum-spanning tree (MST) was generated for MLST and cgMLST profiles, using default settings parameters of Ridom. Missing alleles were ignored in the pairwise comparisons. A neighbour-joining tree was created by pairwise analysis of identified alleles, with missing targets ignored using default settings. The tree and the associated metadata were visualized with iTol v 6.7.2.

### 2.5. Resistome and Virulome Characterization

AMR genes were identified in silico using PointFinder and ABRicate v. 1.0.1 (https://github.com/tseemann/abricate/, accessed on 14 March 2022) by querying the publicly available Comprehensive Antibiotic Resistance Database (CARD) [26,27]. Specific point mutations known to mediate resistance to fluoroquinolones (gyrA), macrolides (23S rRNA) and streptomycin (rpsL), in *C. coli* and *C. jejuni*, were sought in the 542 *Campylobacter* spp. genomes by using the publicly available PointFinder database version. Virulence genes were identified with ABRicate v 1.0.1 (VFDB, updated 19 January 2023) using default minimum coverage and identity settings.

## 3. Results

### 3.1. MLST Analysis of C. jejuni and C. coli Isolates from Italian Poultry

**Figure 1:**
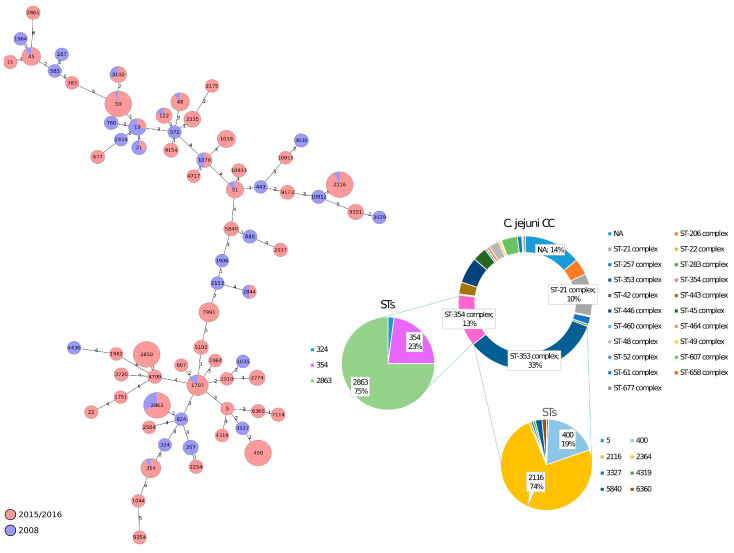
Minimum spanning tree (MST) generated for *C. jejuni* isolates using the MLST approach. MST was calculated by pairwise comparison of 7 target genes with missing values ignored. Nodes correspond to unique profiles and are coloured according to collection date. The connecting lines between STs depict the number of allelic differences between them.

**Figure 2:**
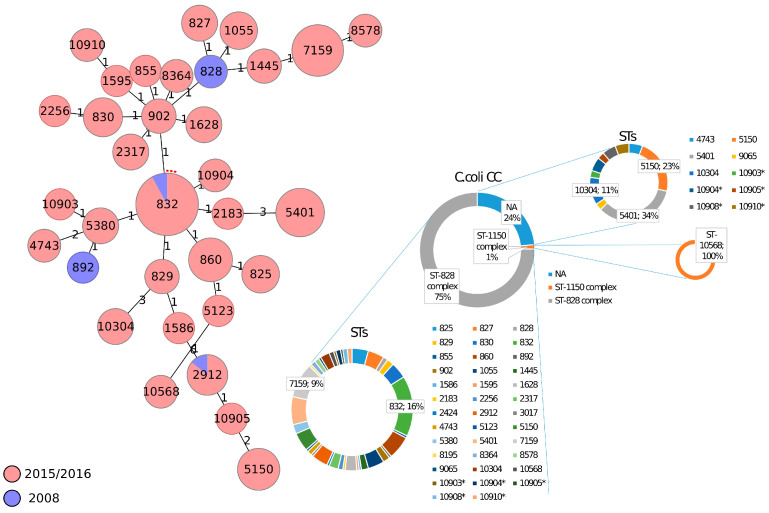
Minimum spanning tree (MST) generated for *C. coli* isolates using the MLST approach. MST was calculated by pairwise comparison of 7 target genes with missing values ignored. Nodes correspond to unique profiles and are coloured according to collection date. The connecting lines between STs depict the number of allelic differences between them. Asterisks (*) refer to new STs found in this work.

The MLST analysis of the 542 *Campylobacter* isolates, from both National Monitoring Plans, is shown in Figure 1 for *C. jejuni* and in Figure 2 for *C. coli*. All *C. jejuni* isolates were classified into 68 different sequence types (STs). Among them, 47 STs belonged to 20 previously characterized clonal complexes (CCs), and the remaining 21 STs were not assigned to any CCs. Newly identified MLST profiles have been added to the *Campylobacter* PubMLST database (https://pubmlst.org/organisms/Campylobacter-jejunicoli/), as shown in Table S1. Three CCs (CC353, CC354 and CC21) were predominant among *C. jejuni* isolates and 56% of the strains were assigned to one of these three CCs (Figure 1). In particular, two *C. jejuni* lineages, ST2116 (CC353) and ST2863 (CC354) dominated, with 74% and 75% of the isolates belonging to the respective CCs (Figure 1), and were isolated at both time-points investigated, indicating their continued widespread presence. CC and ST diversities were observed between the strains isolated in the two different National Plans and between the two isolation sources (carcass skin and caecal content) (Figures S1 and S2). Specifically, 18 STs were only found in *C. jejuni* strains isolated in 2008, while 35 other STs were only found in *C. jejuni* strains isolated in 2015, resulting in a STs overlap of 19.12% (13/68) between the two collection dates considered in the study (Figure 1). CC354, CC257 and CC21 prevalence was higher in *C. jejuni* strains from the 2008 survey with respect to those isolated in 2015 (21%, 8% and 19% vs. 11%, 1% and 9%, respectively) (Figure S1). On the other hand, CC353 showed a consistent prevalence in the 2015–2016 strains with respect to 2008 (38% vs. 13%) (Figure S1). As a consequence, we observed a prevalence of the ST2863 strains in 2008 (17%), compared to that of 2015–2016 (8%) and a prevalence of ST2116 in the 2015–2016 strains (27%), with respect to 2008 (11%) (Figure S1). There was no significant variation in the distribution of most of the CCs between the two isolation sources, except for CC21, which decreased from 29% (2008) to 7% (2015–2016) in strains isolated from the caecal contents, CC354, which decreased from 23% (2008) to 9% (2015–2016) in strains isolated from the caecal contents, and CC353 which, instead, increased from 17% to 43% in strains isolated from the caecal contents and from 11% to 34% in strains isolated from the skin carcasses (Figure S2). Surprisingly, seven CCs (CC22, 283, 42, 460, 49, 52 and 61) were detected only in skin carcasses while one CC was only detected in caecal contents (CC658). Interestingly, ST2116 and ST2863 was predominant in skin carcass samples, with the ST2863 presence significantly higher with respect to caecal content samples (Figure S2).

On the other hand, *C. coli* strains clustered more closely together than *C. jejuni* strains. A total of 162 *C. coli* isolates was divided into 37 distinct STs. CC828 was the dominant clonal complex accounting for 77% (125/162) of all *C. coli* isolates, whereas two isolates with ST10568 were assigned to CC1150 and the remaining STs (35/162) were unassigned (Figure 2). Specifically, CC1150 was assigned only to strains from 2015–2016. The occurrence of ST832, ST828, ST2912 and ST892 was higher in strains from 2008 (29%, 29%, 29%, and 14%) with respect to those from 2015–2016 (15%, 0%, 4%, and 0%). However, one limitation of this study is that the *C. coli* dataset from 2008 was restricted to only seven strains. Finally, only 8.1% (3/37) of *C. coli* STs was shared between the two collection dates considered. Figures S1 and S2 show the number of strains assigned to all the *C. jejuni* CCs and *C. jejuni* STs in the different National Plans and sources analysed in this study.

### 3.2. cgMLST Analysis of C. jejuni and C. coli Isolates from Italian Poultry

**Figure 3:**
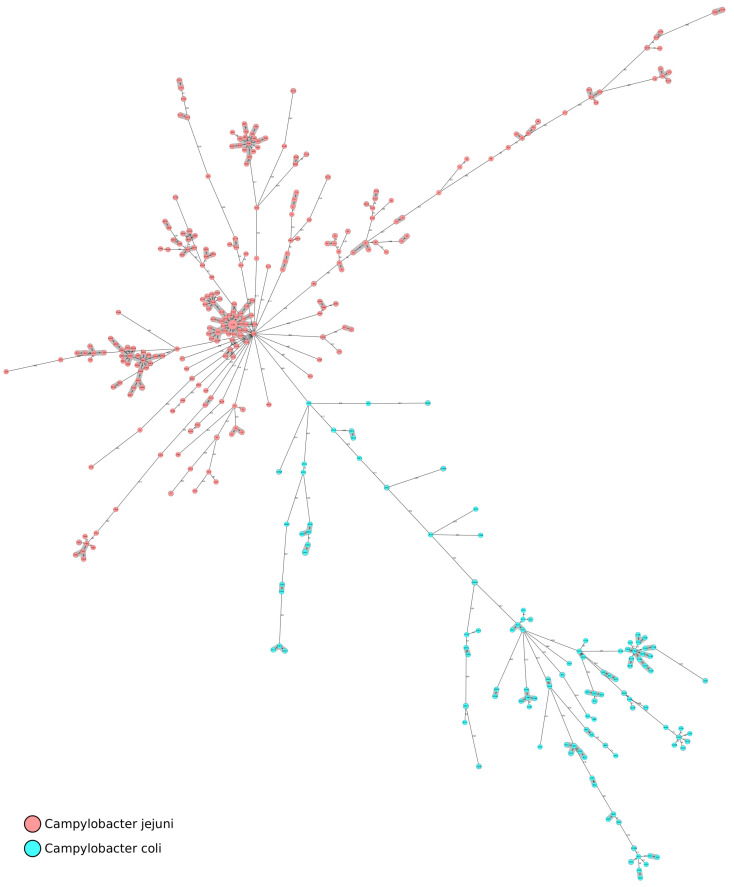
Minimum spanning tree (MST) calculated for 542 *C. jejuni* isolates (indicated in red) and *C. coli* isolates (indicated in green) from Italian poultry, based on cgMLST profiles. The MST was generated with Ridom Seqshere+, ignoring missing values in pairwise comparisons. The circle size is proportional to the genome number for each cgMLST genotype. The branch labels correspond to the number of different alleles between cgMLST profiles.

The cgMLST analysis of 542 genomes of *C. jejuni* and *C. coli* revealed the presence of 15 clusters, setting a cluster distance threshold of 13 alleles (Figure 3). The maximum distance between the pair of cgMLST profiles for *C. jejuni* and *C. coli* was 618 and 584 genes, respectively. The strains from Italian poultry were scattered along most of the branches of the phylogenetic tree. Few clusters of genetically closely related genotypes, 11 for *C. jejuni* and 4 for *C. coli*, could be determined. Interestingly, even within these clusters, we did not note a clear geographic separation, as they often included strains isolated in all the locations analysed. The biggest cluster (1), contained 83 *C. jejuni* samples assigned to ST2116, followed by cluster 2 with 35 samples of *C. jejuni* assigned to ST2863 and by cluster 3, with 19 samples of *C. jejuni* with ST400 (Figure 3). The other *C. jejuni* clusters included nine samples with ST354, eight samples with ST1039, seven samples with ST2850 and ST50, six samples assigned to ST7991 and ST1707 and, finally, five samples assigned to ST3335 and ST1707 (Figure 3). Similarly, *C. coli* isolates were divided into four separate lineages (Figure 3). Cluster 4, the largest, included 10 samples assigned to ST832, followed by cluster 6 (9 samples) assigned to ST5401, cluster 8 (7 samples) assigned to ST7159 and cluster 14 (5 samples) assigned to ST825 (Figure 3).

### 3.3. Comparison of Italian and Global Strains of C. jejuni CC353 and CC354 Isolated from Poultry

**Figure 4:**
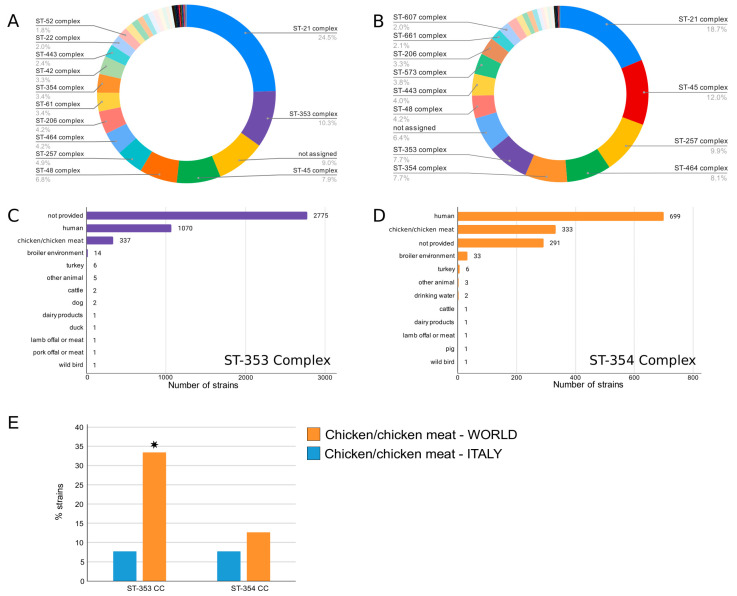
Global distribution of CC353 (ST-353 Complex) and CC354 (ST-354 Complex) in all sources and in chicken/chicken meat. (**A**) = CCs distribution in all sources; (**B**) = CCs distribution in chicken sources; (**C**,**D**) = distribution of CC353 and CC354 in different sources; (**E**) = Distribution of CC353 and CC354 in global and Italian chicken (* *p* < 0.05, X2 test).

The Italian and the global datasets available in the *Campylobacter* database in PubMLST were analysed using cgMLST, focusing on the two most prevalent CCs found in Italy, CC353 and CC354. To select the data to be included in the analysis, the metadata and CCs distribution of *C. jejuni* strains present in the database were examined. Considering the CC prevalence in all sources, our results seem to be in contrast with the global CC distribution in the chicken/chicken meat sources, shown in Figure 4. Particularly, CC21 was the most prevalent at the global level, while it resulted in the third most prevalent CC in our dataset. By contrast, the most prevalent CCs in Italy, CC353 and CC354, were the fifth most prevalent CCs in the global CCs distribution isolated from chicken sources. In particular, 25.4% of strains isolated from human sources and only in 8% of strains isolated from chicken/chicken meat sources belonged to CC353. CC354, instead, was assigned to 51% of strains isolated from humans and 8% of strains isolated from chicken/chicken meat sources. Interestingly, our study showed that CC353 occurrence was statistically higher (33.42%) in Italian poultry strains than in the global distribution in chicken/chicken meat (7.7%), different to that of CC354 that showed similar levels in global (7.7%) and Italian chicken strains (12.63%) (Figure 4). These results are noteworthy because CC353 is one of the most common clonal complexes associated with human disease worldwide.

We performed cgMLST analysis to investigate possible clonal relationship between the strains belonging to CC353 and CC354, using strains from our dataset and the publicly available genomes of isolates collected from poultry sources worldwide. The MST analysis of CC353 showed that most Italian strains were not genetically related to strains from other countries and, with the exception of two STs, were located at a distance of >100 alleles to the closest foreign strain (Figure S3). One Italian isolate (ST2364) was located at a distance of 15 alleles from the neighboring strain from France and the Italian ST400 genomes clustered together with isolates from the United Kingdom, with a minimum distance of 32 alleles between the closest neighboring strains. The most prevalent Italian ST (ST2116) clustered separately from the poultry-associated isolates from the other countries, confirming that this ST was confined to the Italian poultry population. The results of MST analysis of CC354 were similar to our finding for CC353. In general, Italian genomes were found at a distance of more than 100 alleles from the genomes of *C. jejuni* isolated in the other countries (Figure S4). The exception was ST354 that clustered strains from Italy, the United Kingdom, Lithuania and Egypt, suggesting that these strains originated from one source and may have been acquired through the international trade of live poultry or meat products. Interestingly, for both CCs examined we saw limited overlap between the genetic clusters of poultry strains from different countries. Indeed, many of the STs, such as ST6964, ST1489 or ST3510, originated from single countries. As we did not find any closely related genomes for ST2116 and ST2863 in the public dataset from poultry, we expanded our search to other sources. Only six genomes of ST2116 were available (five of them of an acceptable quality) and no genomes of ST2863 were deposited in the pubMLST database. Interestingly, the cgMLST analysis of the ST2116 showed that a genome isolated in the United Kingdom and three genomes from the United States clustered closely together with genomes from Italy with four or fewer alleles of difference (Figure S5). While the source of strains from the United States was unknown, the strain from the United Kingdom was of human origin, suggesting a probable travel-associated infection.

### 3.4. Virulence Determinants in C. jejuni Isolates

**Figure 5:**
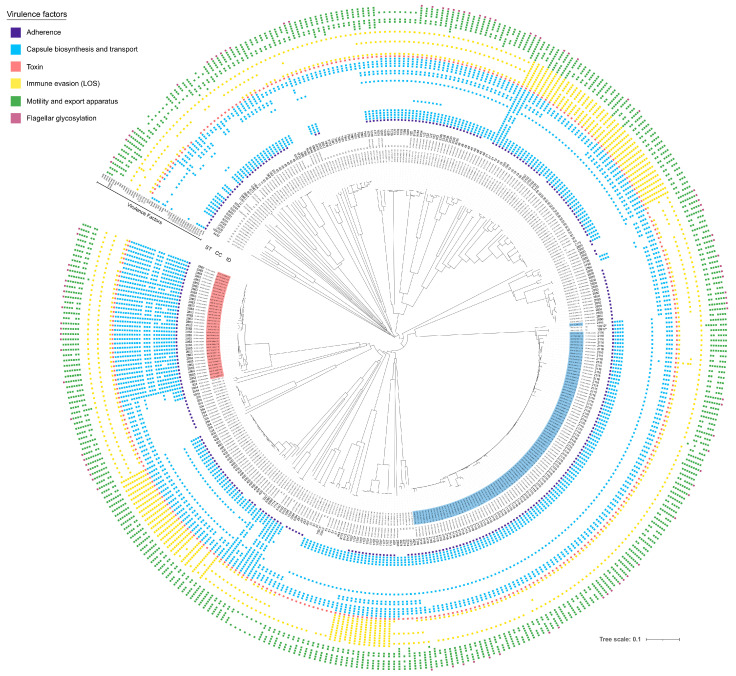
Virulence of *C. jejuni* poultry strains. Phylogenetic tree generated for 380 strains of *C. jejuni* from Italy based on cgMLST performed with RIDOM SeqSphere+ and visualized by iTOL. The presence of AMR genes and allelic diversity of gene substitutions in the analysed genomes are shown in different colours. The phenotypic resistance to antibiotics classes, the National Plans, and strains source origin are annotated. The isolates highlighted in red and in blue belong to ST2863 and ST2116, respectively. Asterisks (*) refer to new STs found in this work.

The 380 *C. jejuni* genomes analysed in this study were grouped in the following three categories: ST2863, ST2116 and the rest of the STs, and tested for the presence of potential virulence genes ordered into six distinct categories (adhesion and colonization, capsule biosynthesis and transport, toxin, immune evasion, motility, flagellar glycosylation) (Table S3) (Figure 5). In total, 119 virulence genes were detected in *C. jejuni* genomes (Table S3). Of these, 60% were present in all genomes examined, while 40% varied between the three selected categories. In particular, the *por*A gene associated with adherence was present in 100% of ST2116 and ST2863 *C. jejuni* strains and in 78% of strains with the other STs. The presence of CDT (cdtA,B,C) encoded by the *cdt*ABC operon was found in almost all strains. Instead, we found differences between the occurrence of genes associated with capsule biosynthesis and transport (Cj1416c; Cj1417c; Cj1419c;Cj1420c; Cj1421c; Cj1422c; Cj1426c; Cj1427c; Cj1432c; Cj1434c; Cj1435c; Cj1436c; Cj1437c; Cj1438c; Cj1440c; *cysC; fcl; glf; hddA; hddC; kfiD; kpsC; kpsE; kpsM; rfbC*) and motility (*flaA; flab ;flgK; pseA; pseD/maf2; pseE/maf5; pseH; ptmA; ptmB*). Interestingly, the number of strains carrying genes associated with capsule synthesis, transport and motility was notably higher in ST2863 isolates than in the other strains in our dataset. Our results showed that the genes associated with capsule biosynthesis and transport and motility were found, respectively, in 91% and 95% of ST2863 isolates, 20% and 81% of ST2116 isolates and 34% and 65% of the rest of the genomes. Moreover, genes associated with the synthesis of LOS were detected as follows: 27% in ST2863, 12% in ST2116 and 30% in the other STs. The virulence genes detected are listed in Table 1.

### 3.5. Antimicrobial Susceptibility

**Figure 6:**
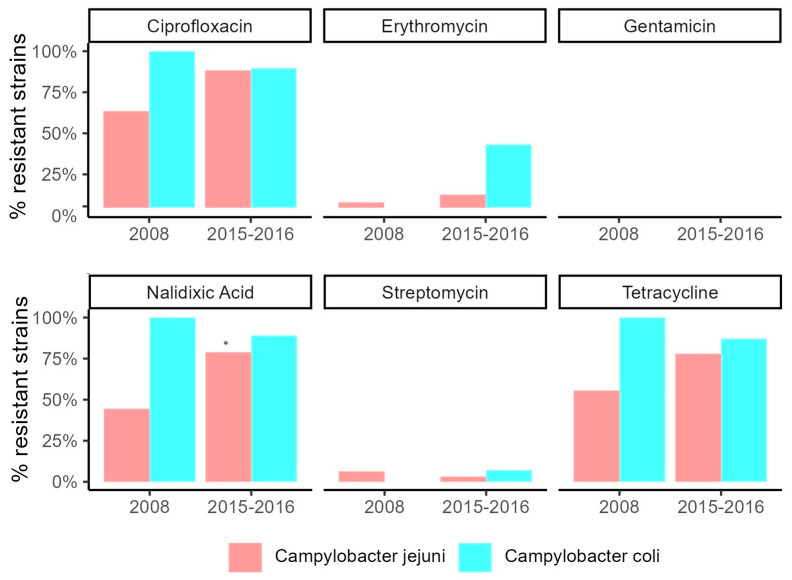
Antimicrobial resistance patterns for all *Campylobacter* isolates. (* *p* < 0.05, X2 test).

**Figure 7:**
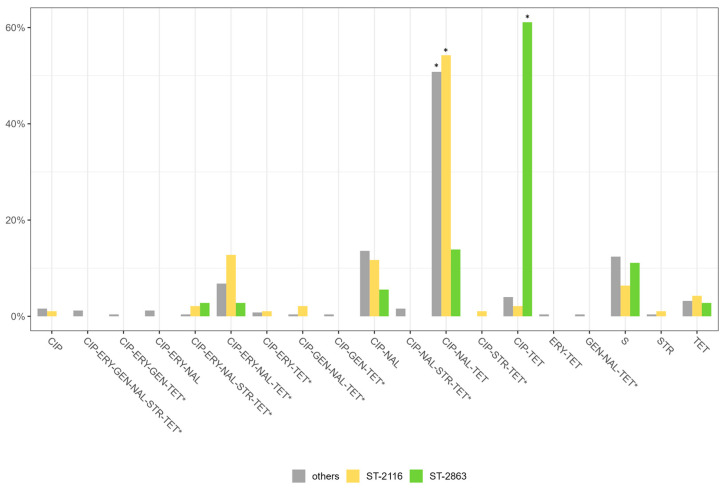
Percentage of resistant and multi-resistant Campylobacter strains according to ST2116, ST 2863 and others. The profiles with an asterisk refer to MDR strains. * *p* < 0.05 X2-test.

The antimicrobial resistance profiles of the *C. jejuni* and *C. coli* are shown in Figure 6. The majority of *Campylobacter* isolates were resistant to ciprofloxacin, nalidixic acid, and tetracycline and differences were observed between *C. jejuni* and *C. coli,* as well as between the years of isolation (Figure 6 and Figure S6). In particular, among the *C. jejuni* collected in 2008, 63.5% were resistant to ciprofloxacin, 44.4% to nalidixic acid and 55.6% to tetracycline. Conversely, low numbers of resistant strains were observed for erythromycin (7.9%), gentamicin (1.6%) and streptomycin (6.3%). Among *C. jejuni* collected in 2015, higher proportions of resistant strains were observed for ciprofloxacin (88%), nalidixic acid (79%) and tetracycline (78%). Similarly to previously isolated strains, low resistance levels were found for erythromycin (13%), gentamicin (3%) and streptomycin (3%). Among *C. coli* isolated in 2008, all strains were resistant to ciprofloxacin, nalidixic acid and tetracycline, while 90% of *C. coli* collected in 2015 was resistant to ciprofloxacin, 89% to nalidixic acid, 87% to tetracycline and 43% to erythromycin. Very low levels of resistance were found for gentamicin and streptomycin, 1% and 7%, respectively. Comparing the two periods, we observed a statistically significant increase only for nalidixic acid in *C. jejuni* strains isolated in 2015–2016, while a notable increase in erythromycin resistance was demonstrated for *C. coli* strains of the most recent collection (Figure 6). MDR, regarded as the resistance to at least three different classes of antibiotics [1], is shown in Figure S6. The frequency of MDR isolates was higher in *C. coli* (47%) collected in 2015–2016 than in the other groups. Among *C. jejuni* strains isolated in 2008 and 2015–2016, 11% and 14% were , respectively; while in *C. coli* isolates, no MDR strains were found in 2008. The most common MDR profile found (53.3% of *Campylobacter*) included ciprofloxacin, erythromycin, nalidixic acid and tetracycline (4.76% and 8.52% of *C. jejuni* strains collected in 2008 and 2015–2016, and 40% of *C. coli* strains isolated, respectively, in 2015). Furthermore, few isolates showed resistance to four different classes of antibiotics (quinolones, erythromycin, aminoglycosides and tetracycline). Four MDR patterns were detected in both species. Figure S6 shows the MDR observed in this study. In addition, comparing the predominant STs (ST2116 and ST2863) with the other STs obtained in the study, ST2116 (54%) showed a higher resistance to quinolones, fluoroquinolones and tetracycline with respect to ST2863 (14%) (Figure 7). By contrast, ST2863 *C. jejuni* strains showed a significant increase, with respect to the other two groups investigated, for tetracycline and ciprofloxacin (Figure 5). MDR (to quinolones, fluoroquinolones, macrolides and tetracycline) was observed in 13% of the ST2116 *C. jejuni* strains with respect to 3% of the ST2863 *C. jejuni* strains and 7% of the other STs (Figure 7).

### 3.6. Antimicrobial Resistance Genes

Table S2 lists the genetic AMR determinants investigated in this study. The selected AMR genes were evaluated exclusively in all *Campylobacter* isolates phenotypically resistant to the tested antimicrobials. In particular, 10 acquired genes (*tet*(O), *bla*OXA−184, *bla*OXA−450, *bla*OXA−61, *bla*OXA−193, *bla*OXA−605, *aadE*, *aph(3′)-IIIa*, *aad9*, *sat4*) and 3 point mutations (in *gyrA*, *rpsL*, and 23S rRNA) associated with resistance to 5 distinct antibiotic classes were identified. Furthermore, *cmeA*, *cmeB*, *cmeC*, *cmeR* genes responsible for pump efflux mechanisms were also investigated. Among tetracycline-resistant *Campylobacter*, most strains collected in 2008 and 2015–2016 harboured the *tet*(O) gene, showing concordance rates between phenotypic and genotypic resistances ranging from 87% to 100% (Table S2). The resistance-associated point mutation (T86I) in the quinolone-resistance-determining regions (QRDR) of the *gyrA* gene was detected in the majority of the strains resistant to quinolones, with percentages of concordance ranging from 90% to 100% (Table S2). The erythromycin-resistant strains were examined for the presence of mutation (A2075G) in the 23S rRNA gene, showing a concordance rate of 20% for *C. jejuni* and of 94% for *C. coli* collected in 2015. Even though we did not test resistance to β-lactam antibiotics phenotypically, we detected high percentages of genes associated with β-lactam resistance (particularly *bla*OXA-450 gene present in all *Campylobacter*, ranging from 60% to 87%). By contrast, some genes were mainly associated with *C. coli* strains. These included *bla*OXA-61 (71% and 92%, in *C. coli* collected in 2008 and 2015, respectively); *bla*OXA−193 (43% and 69%, in *C. coli* collected in 2008 and 2015, respectively) and *bla*OXA−605 (57% and 91%, in *C. coli* collected in 2008 and 2015, respectively). Our analysis also revealed one additional putative OXA-type β-lactamase gene, *bla*OXA-184 which is found only in *C. jejuni* strains, showing, however, a lower prevalence (9.5% and 14%). In addition to genes for β-lactamases, we found that 7 strains out of 11 streptomycin-resistant strains harboured the *aadE* gene, associated with streptomycin resistance, showing a concordance rate of 64%. The *cmeA, cmeB, cmeC and cmeR* genes, associated with the *Campylobacter* multidrug efflux complex, were present in almost all strains of both species. No strains harboured genes associated with gentamicin resistance, as added to which no amino acid substitutions in the RpsL, involved in streptomycin resistance, was found. A phylogenetic tree generated for *C. jejuni* and *C. coli* strains based on cgMLST, is shown in Figures S7 and S8.

## 4. Discussion

In this study, we analysed a *Campylobacter* population from poultry to monitor antimicrobial and molecular changes over a decade in Italy. Our study revealed a high molecular diversity of *Campylobacter* isolated from chicken sources and demonstrated the persistence of two particular *C. jejuni* STs (ST2116 and ST2863) persistent in Italian poultry over the period. These two STs are associated with CCs, which are widespread in chicken and in samples of human stools in Italy, as well as in other countries (http://pubmlst.org/Campylobacter) [28,29]. Notably, while ST2116 and ST2863 are frequently isolated in Italy, only 35 and 32 isolates, respectively, have been reported in pubMLST so far (accessed on 4 April 2022). Of these, only 27 isolates from chicken (27/35, 77.14%) and 8 from human stools (8/35, 22.85%) were identified as ST2116; while 22 isolates from chicken (22/32, 68.75%), 10 from human stools (10/32, 31.25%) and 1 from a pig (1/32, 3.12%) were classified as ST2863. Our results seem to be in agreement with data previously reported by other authors [6,28,29], suggesting the existence of local *C. jejuni* clones circulating in restricted geographical areas. These strains may be able to survive better than the others under environmental stress conditions, such as those existing along the poultry food production chain [6]. The genetic or phenotypic determinants responsible for the increased prevalence of some MLST lineages of *C. jejuni* are not yet known; however, they would seem to depend on various factors related to climate, host or geography [29].

Data from this study demonstrated the rapid emergence of two resistant lineages of *C. jejuni* among poultry isolates in Italy from 2008 onwards, indicating also how quickly national antimicrobial resistance levels can change with the introduction of a successful bacterial clone. Furthermore, CC353 and CC354 distribution in the Italian *Campylobacter* population resulted higher compared with global distribution, with CC354 being the more dominant ST in Italian poultry carcasses with respect to faeces. This study also showed that the proportion of these two clones, which are probably the most successfully adapted clones, changed over time. In particular, ST2863 *C. jejuni* strains decreased from 17.46% (2008) to 7.8% (2015–2016) and ST2116 *C. jejuni* increased in the poultry population from 11.11% (2008) to 27.44% (2015–2016). Furthermore, the genomic proximity of the strains of the same ST would indicate a clonal expansion of ST2116 and ST2863 in the Italian broiler population. In addition, when clustering our isolates by cgMLST with isolates of diverse geographical origins, we found that all CC353 and CC354 Italian poultry genomes clustered separately from r genomes from different countries, suggesting a probable national segregation. Interestingly, two Italian clones distant by only 15 and 19 alleles from the other European clones were identified in France and in Lithuania. These findings would suggest that travel and trade are some of the most important sources of human campylobacteriosis. Our results showed that ST2116 clones from poultry clustered together with ST2116 clones isolated from human samples in England, which suggested that these were travel-acquired cases of *Campylobacter*, rather than cases acquired from the locally farmed broiler. Finally, an in silico screen of the presence of genes linked to virulence factors was performed. We detected a total of 119 virulence genes our dataset. Most of these genes (60%) were conserved in all isolates and potentially involved in causing disease in humans [30,31]. Notably, ST2116 and ST2863 isolates harboured more motility genes than those belonging to other STs. Moreover, many genes associated with capsule synthesis were frequently identified in ST2863 isolates (91%9). These genes have previously been reported to be associated with colonization [17], explaining, in part, the evolutionary success seen for this emergent clone in the poultry population at the national level.

Antimicrobial drugs had been extensively administered in animal husbandry for many years, before being banned in Europe in 2006. However, their overuse in food-producing animals has led to the spread of MDR strains and consequently to the reduced number of treatment options for immunocompromised and severely ill patients [32,33,34,35,36]. Mourkas et al. recently demonstrated the ability of *C. coli* to acquire erythromicyn-resistance genes from other bacteria [13]. Among antimicrobials, macrolides (erythromycin and azithromycin) are the drugs of first choice for the treatment of campylobacteriosis, while fluoroquinolones (ciprofloxacin) and tetracycline are used as an alternative therapy for cases of traveller’s diarrhoea or gastroenteritis [21,37,38]. Aminoglycosides drugs, such as gentamicin and streptomycin, are instead usually used in case of severe bacteraemia [39]. A very high prevalence of resistance to fluroquinolones and tetracycline was observed in this study, considering the great risk of the current isolates to public health. In particular, an increase in the resistance of *C. jejuni* isolated in 2015–2016 with respect to those isolated in 2008 was detected. Moreover, in *C. coli*, very high and stable resistance levels against these molecules were detected. These results were associated with the detection of the T86I mutation in the *gyrA* in both *C. jejuni* and *C. coli* and the occurrence of the *tet(*O) gene, present in almost all tetracycline-resistant *C. jejuni* isolates. Indeed, the concordance rates for phenotypic and genotypic resistance were, respectively, around 86–100% for tetracycline and 90–100% for ciprofloxacin and nalidixic acid.

*Campylobacter* tetracycline-resistant strains are widespread [40]. Tetracycline is commonly used in the poultry industry, and serves as an important reservoir of resistant strains [41]. A worrying increment in erythromycin resistance was detected in *C. coli* isolated in 2015–2016, confirmed by the mutation in the 23S rRNA gene. No resistance to erythromycin in *C. coli* isolated in 2008 was found. This, however, could be due to the very low number of strains included in our dataset.

Importantly, we observed a high prevalence of MDR strains among *C. coli* isolates simultaneously resistant to ciprofloxacin, erythromycin, nalidixic acid and tetracycline that was detected in 40% of strains. The findings of the current study are consistent not only with the general trends in *Campylobacter* antibiotic susceptibility in poultry in Europe, but also worldwide [3,42]. By contrast, some countries such as Australia [43] and Northern European countries [3,44] reported much lower resistance levels to fluoroquinolones. These may reflect the absence of or limitation in the use of those antimicrobials in livestock or a greater attention to biosecurity and biosafety measures applied on farms. For this reason, global differences in the use of antimicrobials are also likely responsible for the geographical differences observed in antimicrobial resistance. The results of this study provide critical insights into the levels of antibiotic resistance in *C. jejuni* and *C. coli* from Italian poultry, which could contribute to the development of surveillance programs of antimicrobial resistance in Italy. Altogether, the current study provided genomic data on the diversity and resistance of a large collection of *C. jejuni* and *C. coli* from Italian poultry. Further studies are needed to determine genes associated with virulence and quantify accessory genomes shared among the different lineages, which may be linked to the spread of AMR.

## 5. Conclusions

Poultry meat is a major source of *Campylobacter* and it has been linked to a large number of outbreaks and sporadic cases of the disease. The consumption of poultry meat in the EU varies by country, but poultry is a popular source of protein in many EU countries, including Italy. The consumption of poultry meat is therefore a critical factor in the spread of *Campylobacter* and the incidence of campylobacteriosis. The worrying increase in antibiotic resistance, observed in *Campylobacter* strains isolated from Italian poultry, highlights the potential risk of the spread of these resistant strains to the human population through the food chain. The prevalence of the most virulent strains revealed in this study suggests that these strains may be adapted to the poultry environment and may pose a significant risk to consumers. The emergence of MDR instead highlights the necessity to support the design of surveillance programs and to implement control measures for the use of antimicrobials at the farm level. To minimize the emergence of *Campylobacter* resistance it is vital to follow common policies and implement good practices on antimicrobial application in animal production. Resistant bacteria in the food production chain can easily be transmitted to the consumer, causing a serious risk to public health. The implementation of good practices on biosecurity and antimicrobial usage at the farm level is crucial to minimize the emergence of resistance and prevent the spread of resistant pathogens in the food production chain, ultimately protecting public health.

## Figures and Tables

**Table 1:** Virulence factors in *C. jejuni* isolates from poultry.

	Adherence	Toxin CDT			Flagellar Glycosylation	Capsule Biosynthesis and Transport	Immune Evasion	Motility
STs	porA	cdtA	cdtB	cdtC	maf4	no. 25 ^a^	no. 9 ^b^	no. 9 ^c^
2116 (n = 94)	100%	100%	100%	100%	20%	20%	12%	81%
2863 (n = 36)	100%	97%	100%	100%	39%	91%	27%	95%
Others (n = 250)	78%	99%	99%	100%	11%	34%	30%	65%

^a^ Number and list of genes associated with capsule bio-synthesis and transport (Cj1416c; Cj1417c; Cj1419c; Cj1420c; Cj1421c; Cj1422c; Cj1426c; Cj1427c; Cj1432c; Cj1434c; Cj1435c; Cj1436c; Cj1437c; Cj1438c; Cj1440c; cysC; fcl; glf; hddA; hddC; kfiD; kpsC; kpsE; kpsM; rfbC); ^b^ Number and list of genes associated with immune evasion (Cj1135; Cj1136; Cj1137c; Cj1138; cstIII; gmhA2; neuA1; neuB1; neuC1); ^c^ Number and list of genes associated with motility (flaA; flaB; flgK; pseA; pseD/maf2; pseE/maf5; pseH; ptmA; ptmB).

## Data Availability

The data used to support the findings of this study can be made available by the corresponding author upon request.

## Supplementary Materials

The following supporting information can be downloaded at: https://www.mdpi.com/article/10.3390/foods12152919/s1, Figure S1: *Campylobacter jejuni* CCs and STs prevalence according to National Plans 2008 (A–C) 2015/2016 (B,D); Figure S2: *Campylobacter jejuni* CCs distribution in carcasses and faeces according to National Plans 2008 (A), 2015/2016 (B) and combined (C). ST2116 and ST2863 distribution in carcass and faeces according the two National plans (D). * *p* < 0.05 X2-test; Figure S3: MST of *C. jejuni* CC353 poultry isolated from 10 different countries based on cgMLST profiles. The branch labels correspond to the number of discriminative alleles; Figure S4: MST of *C. jejuni* CC354 poultry isolated from 10 different countries based on cgMLST profiles. The branch labels correspond to the number of discriminative alleles; Figure S5: Downloaded genomes of ST2116 (genomes of 2864 absent in pubMLST DB)—6 scaffolds downloaded, one excluded for poor Q; performed cgMLST + MST for the 5 world genomes versus Italian ST2116; Figure S6: Percentage of *C. jejuni* and *C. coli* strains from poultry displaying different antimicrobial susceptibility levels (S = sensitive, R = resistant to one, or more molecules of the same class of antimicrobials ; MDR = multidrug resistant to three antimicrobial molecules of a different class); Figure S7 CgMLST analysis of *C. jejuni* poultry strains. Phylogenetic tree generated for 380 strains of *C. jejuni* from Italy is based on cgMLST performed with RIDOM SeqSphere and visualized by iTOL. The presence and allelic diversity of antimicrobial resistance genes or substitutions in *C. jejuni* genomes are indicated in different colours as well as phenotypic resistance to 1–5 antibiotics classes, National Plans and strains source origin. The isolates highlighted in red and in blue belonging to ST2863 and ST2116, respectively; Figure S8 CgMLST analysis of *C. coli* poultry strains. Phylogenetic tree generated for 162 strains of *C. coli* from Italy is based on cgMLST performed with RIDOM SeqSphere and visualized by iTOL. The presence and allelic diversity of antimicrobial resistance genes substitutions in *C. coli* genomes are indicated in different colours as well as phenotypic resistance to 1–5 antibiotics classes, National Plans and strains source origin; Table S1. List of poultry isolates with MLST and AMR genes profiles, phenotypic resistance and mutations; Table S2. Genotypic resistance to antibiotics in *C. jejuni* and *C. coli* isolated from Italian poultry.
