# Supplementary material for: Genomic and Antimicrobial Surveillance of Campylobacter Population in Italian Poultry
Source: Foods. 2023 Jul 31;12(15):2919. doi: 10.3390/foods12152919 (PMC10418777; doi:10.3390/foods12152919)
Supplement: Supplementary file 1 [file foods-12-02919-s001.zip › Figure S8.pdf]

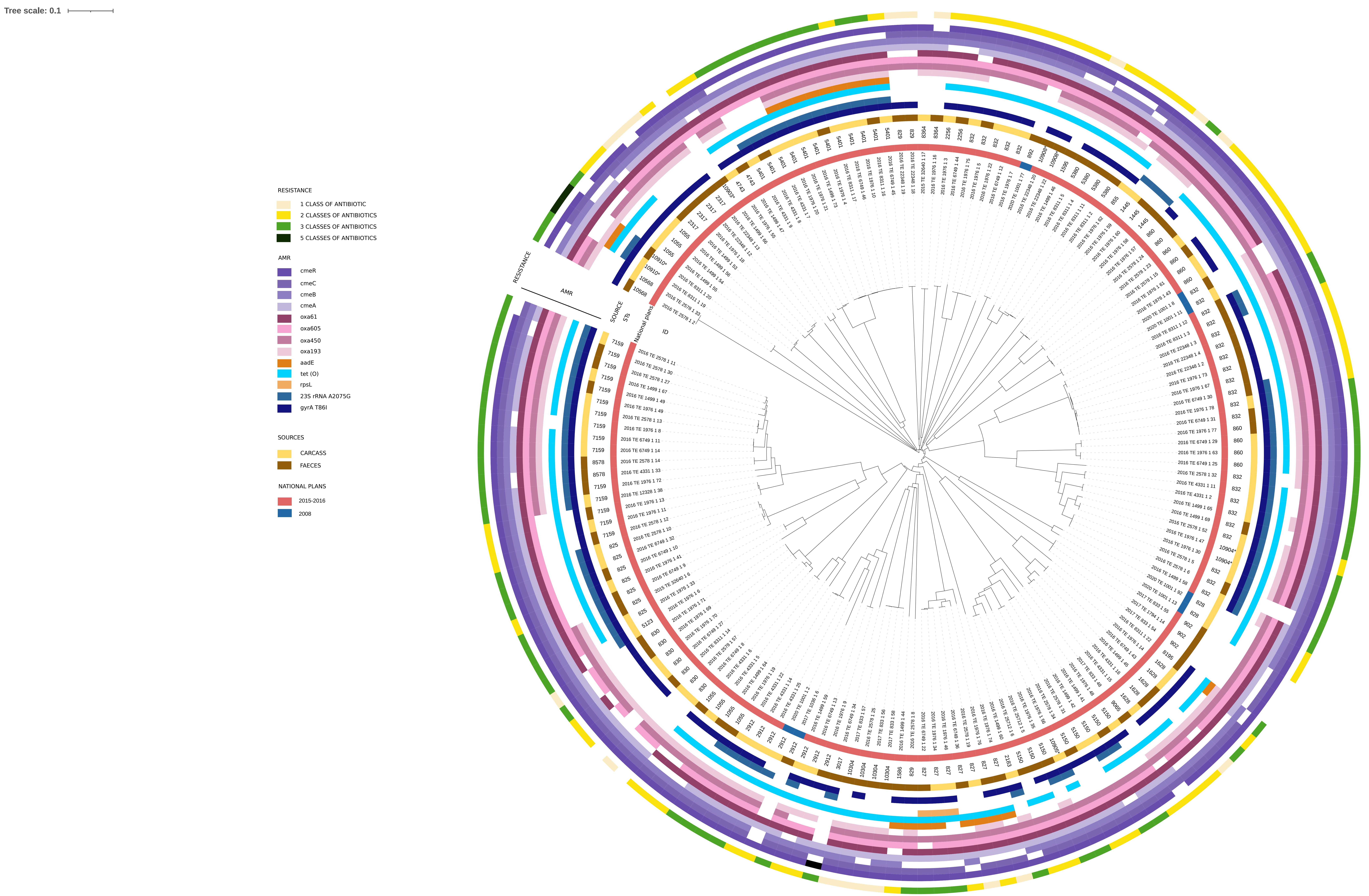

Tree scale: 0.1
RESISTANCE
1 CLASS OF ANTIBIOTIC
2 CLASSES OF ANTIBIOTICS
3 CLASSES OF ANTIBIOTICS
5 CLASSES OF ANTIBIOTICS
AMR
cmeR
cmeC
cmeB
cmeA
oxa61
oxa605
oxa450
oxa193
aadE
tet (O)
rpsL
23S rRNA A2075G
gyrA T86I
SOURCES
CARCASS
FAECES
NATIONAL PLANS
2015-2016
2008
SOURCE
STs
National plans
ID
