# Supplementary material for: Genomic and Antimicrobial Surveillance of Campylobacter Population in Italian Poultry
Source: Foods. 2023 Jul 31;12(15):2919. doi: 10.3390/foods12152919 (PMC10418777; doi:10.3390/foods12152919)
Supplement: Supplementary file 1 [file foods-12-02919-s001.zip › Table S2.pdf]

Table S2. Genotypic resistance to antibiotics in *C. jejuni* and *C.coli* isolated from Italian poultry.

Antimicrobial class	Drug	Genetic AMR determinant (gene symbol)	Description	*C. jejuni* (C.d. 2008) concordance rate (%)	*C. jejuni* (C.d. 2015) concordance rate (%)	*C. coli* (C.d. 2008) concordance rate (%)	*C. coli* (C.d. 2015) concordance rate (%)
Tetracyclines	Tetracycline	*tet(O)*	Acquired AMR gene	31/35 (88.6)	214/247 (86.6)	7/7 (100)	121/135 (88.88)
Quinolones	Ciprofloxacin<br>Nalidixic acid	gyrA T86I	Point mutation	40/42 (95.2) cip;<br>27/28 (96.4) NA	228/250 (90.71) cip;<br>231/253 (91.2) NA	7/7 (100) cip;<br>7/7 (100) NA	125/139 (89.9) cip; 124/137 (90.5) NA
β-Lactams		blaOXA−184	Acquired AMR gene	6/63 (9.52)	46/317 (14.5)	-	-
		blaOXA−450	Acquired AMR gene	48/63 (76.2)	189/317 (59.6)	5/7 (71.4)	135/155 (87)
		blaOXA−61	Acquired AMR gene	1/63 (1.6)	8/317 (2.5)	5/7 (71.4)	143/155 (92.2)
		blaOXA−193	Acquired AMR gene	-	-	3/7 (42.8)	107/155 (69)
		blaOXA−605	Acquired AMR gene	-	-	4/7 (57.1)	141/155 (90.9)
Aminoglycosides	Streptomycin	aadE (ant(6)-Ia)	Acquired AMR gene	-	-	-	7/11 (63.6)
	Gentamicin	aph(3′)-IIIa	Acquired AMR gene	-	-	-	-

Macrolides	Erytromycin	23S rRNA A2075G	Point mutation	1/5 (20)	8/40 (20)	-	63/67 (94.02)
Multidrug efflux transporter	Broad spectrum antibiotics	cmeA<br>cmeB<br>cmeC		63/63 (100)<br>53/63 (84.1)<br>63/63 (100)	317/317(100)<br>262/317 (82.6)<br>317/317 (100)	7/7 (100)<br>7/7 (100)<br>8/7 (87.5)	137/155 (88.4)<br>139/155 (89.7)<br>153/155 (98.7)
		cmeR		63/63 (100)	317/317(100)	7/7 (100)	152/155 (98)
