# Supplementary figures and images for: Genomic and Antimicrobial Surveillance of Campylobacter Population in Italian Poultry
Source: Foods. 2023 Jul 31;12(15):2919. doi: 10.3390/foods12152919 (PMC10418777; doi:10.3390/foods12152919)

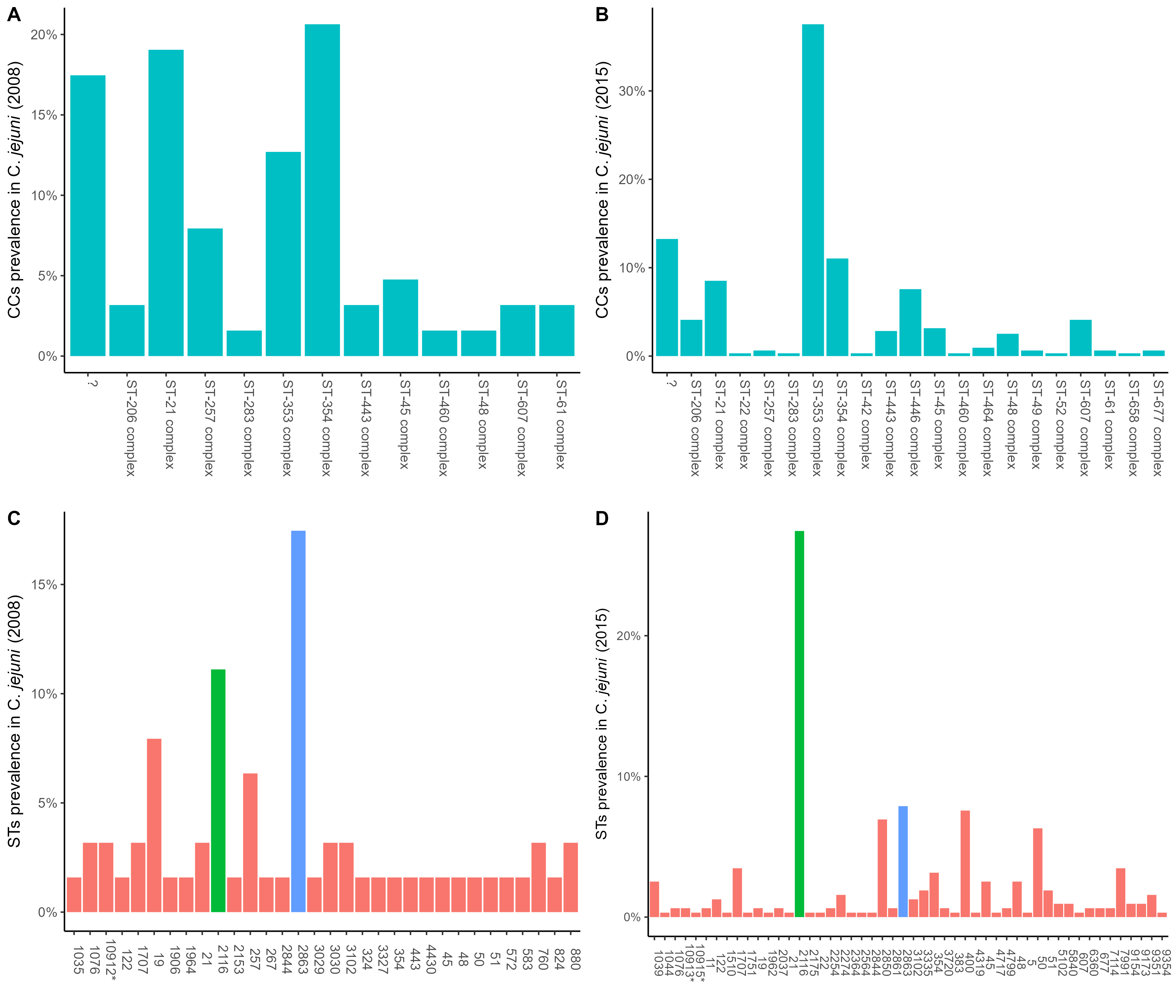

Supplement: Supplementary file 1 [file foods-12-02919-s001.zip › Figure S1.tiff]

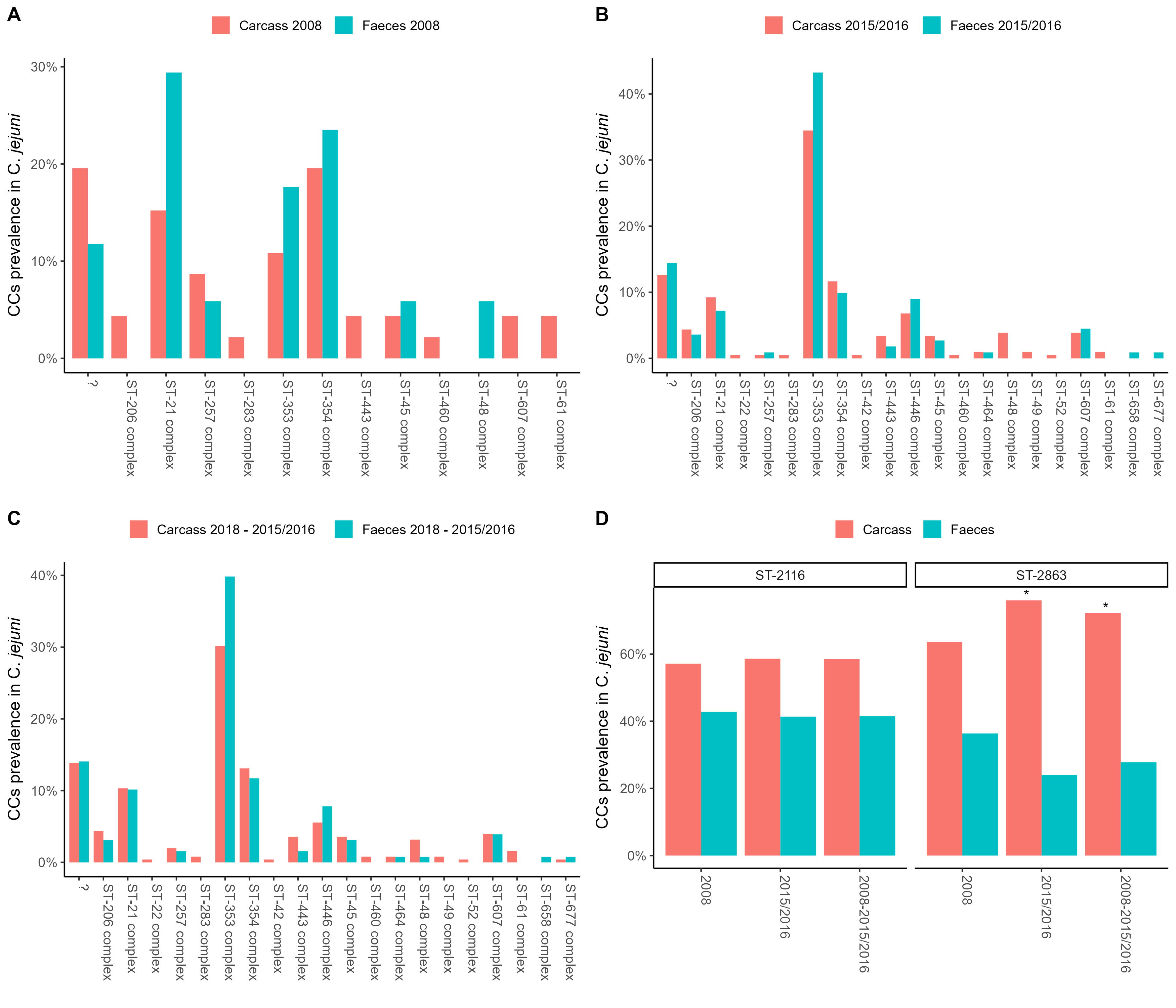

Supplement: Supplementary file 1 [file foods-12-02919-s001.zip › Figure S2.tiff]

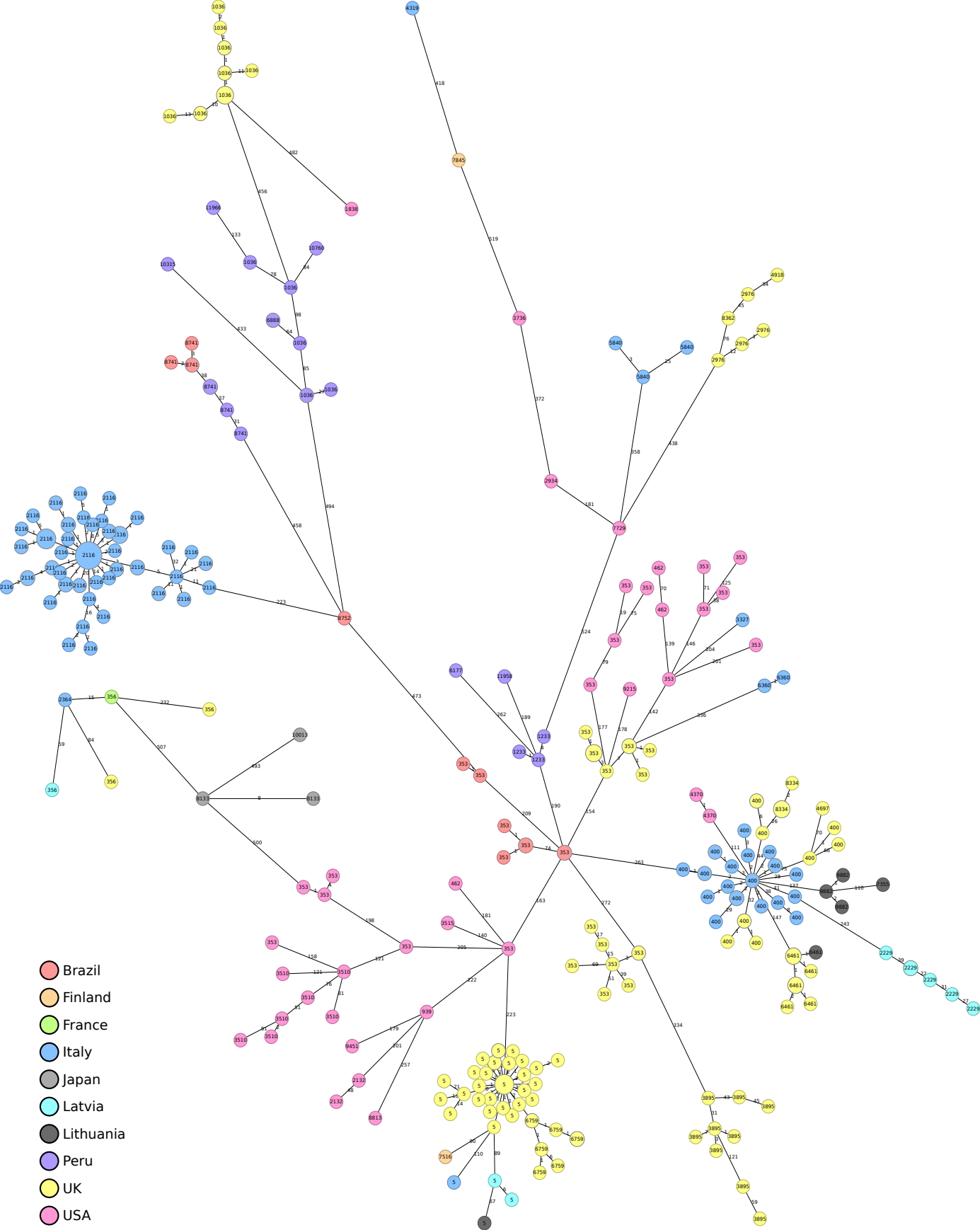

Supplement: Supplementary file 1 [file foods-12-02919-s001.zip › Figure S3.pdf]

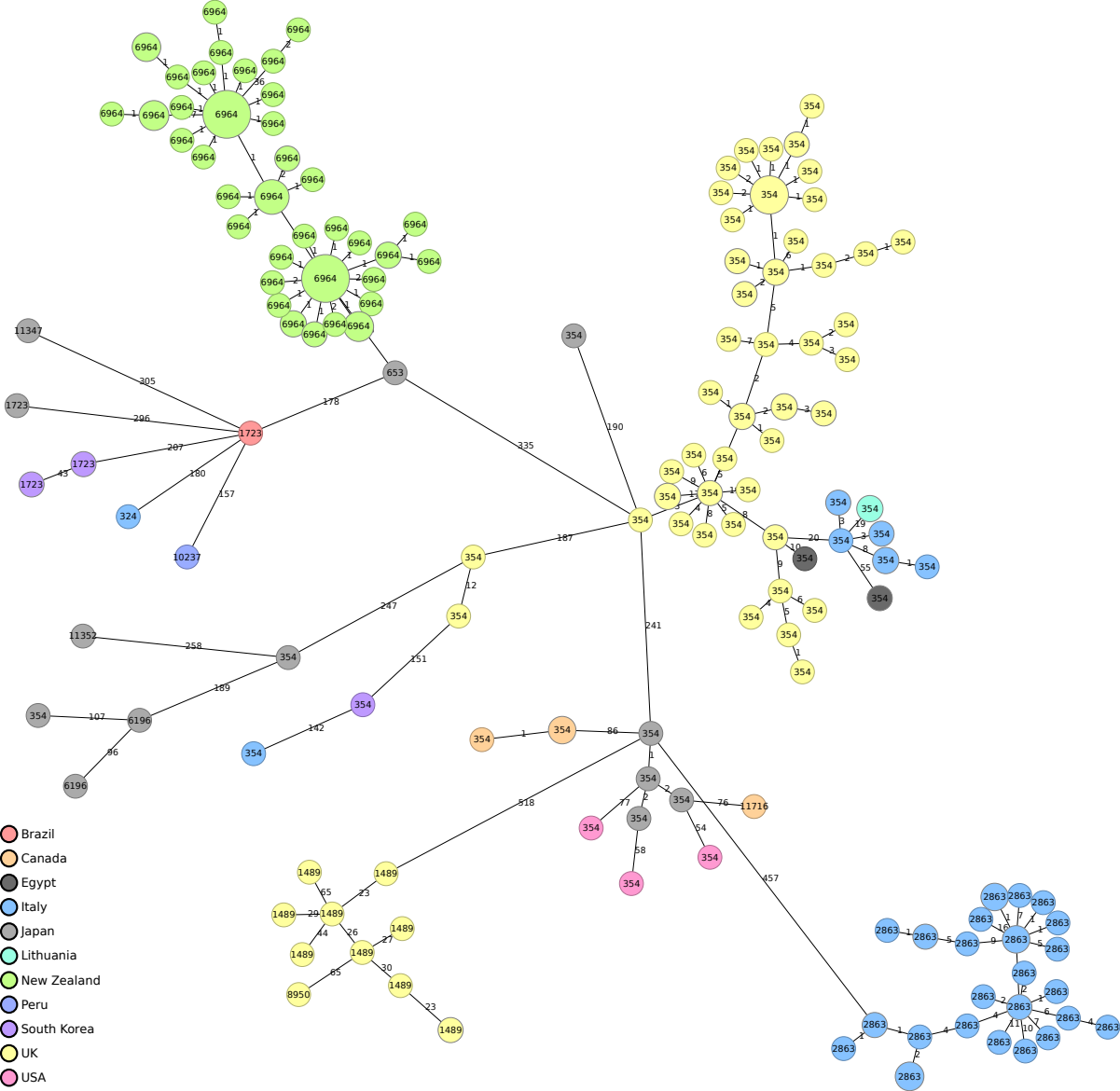

Supplement: Supplementary file 1 [file foods-12-02919-s001.zip › Figure S4.pdf]

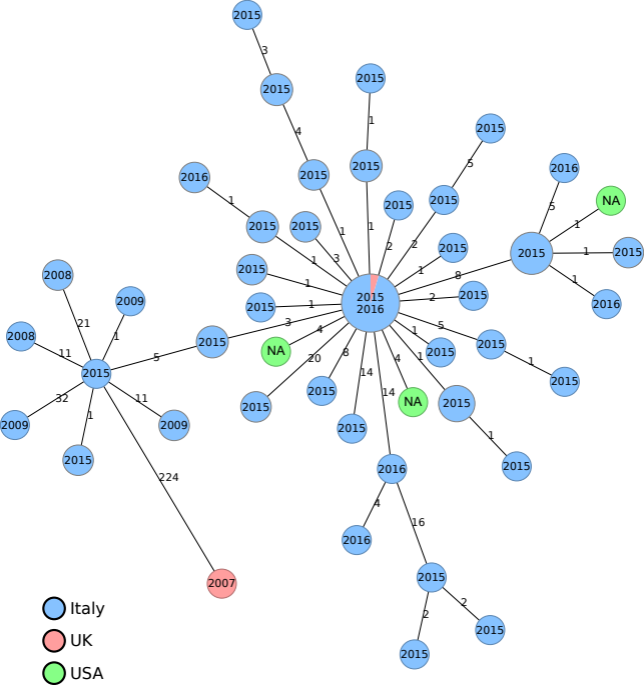

Supplement: Supplementary file 1 [file foods-12-02919-s001.zip › Figure S5.pdf]

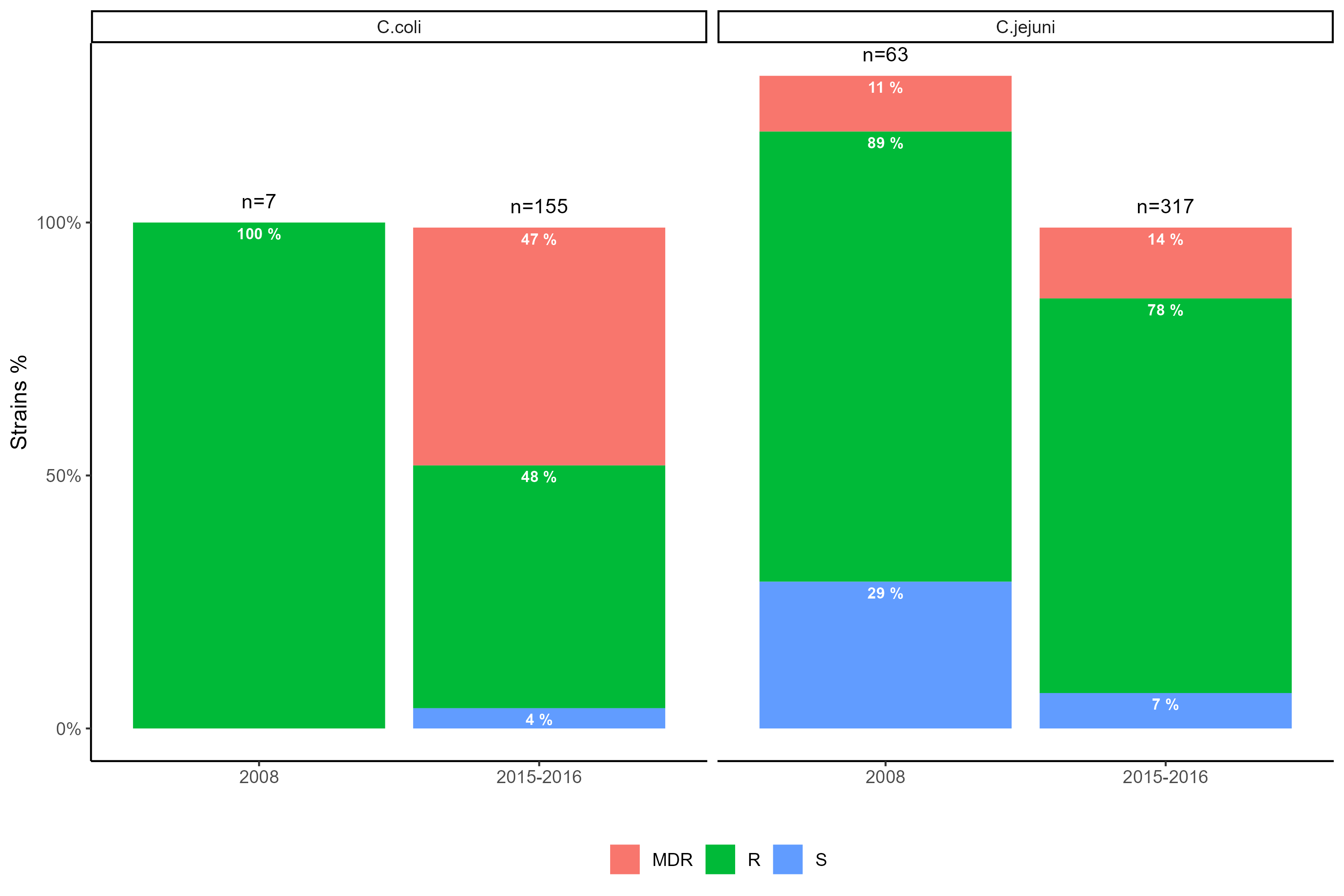

Supplement: Supplementary file 1 [file foods-12-02919-s001.zip › Figure S6.tiff]

Tree scale: 0.1

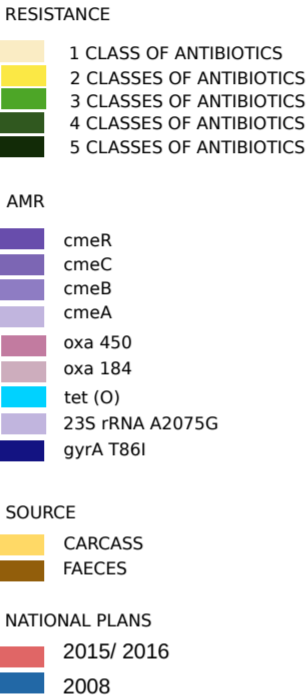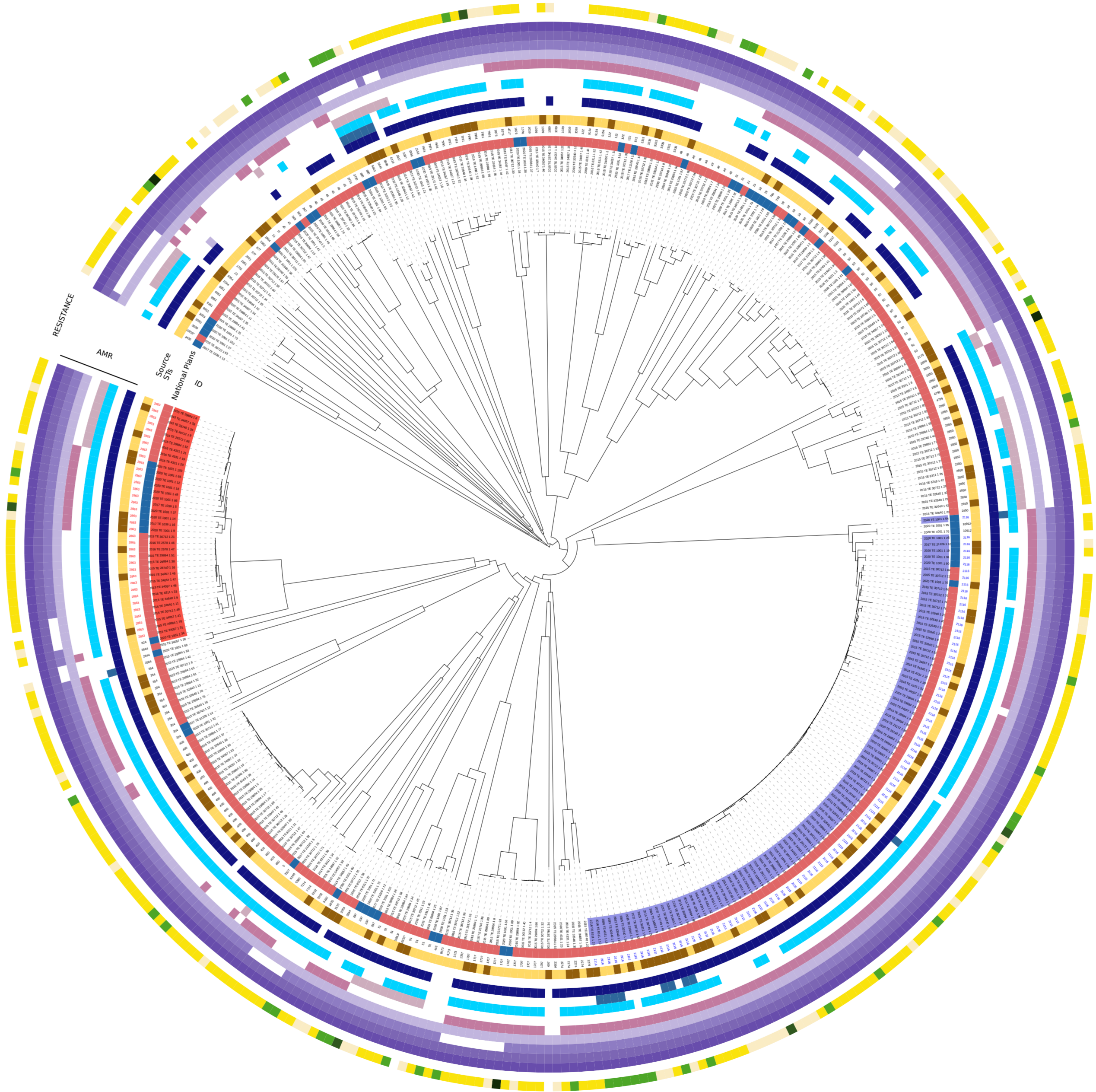

Supplement: Supplementary file 1 [file foods-12-02919-s001.zip › Figure S7.pdf]
